# 2D–3D graphene-coated diatomite as a support toward growing ZnO for advanced photocatalytic degradation of methylene blue†

**DOI:** 10.1039/d1ra07708b

**Published:** 2021-11-30

**Authors:** Xingjian Dai, Hao Zeng, Chuan Jin, Jinsong Rao, Xiaoying Liu, Kailin Li, Yifan Zhang, Yaolun Yu, Yuxin Zhang

**Affiliations:** College of Materials Science and Engineering, Chongqing University Chongqing PR China 400044 Daixingjian@cqu.edu.cn +86 23 65104131; College of Materials Science and Engineering, Chongqing University Chongqing PR China 400044 zengh404@foxmail.com; College of Science, Chongqing University of Posts and Telecommunications Chongqing 400065 PR China jingchuan@cqupt.edu.cn; College of Materials Science and Engineering, Chongqing University Chongqing PR China 400044 rjs@cqu.edu.cn; Engineering Research Center for Waste Oil Recovery Technology and Equipment of Ministry of Education, Chongqing Key Laboratory of Catalysis and New Environmental Materials, College of Environment and Resources, Chongqing Technology and Business University Chongqing 400067 PR China lxy_ctbu@163.com; College of Materials Science and Engineering, Chongqing University Chongqing PR China 400044 likailin920809@163.com; College of Materials Science and Engineering, Chongqing University Chongqing PR China 400044 zhangyifan@cqu.edu.cn; Qian Xuesen Laboratory of Space Technology, China Academy of Space Technology Beijing 100094 China yuyaolun@qxslab.cn; College of Materials Science and Engineering, Chongqing University Chongqing PR China 400044 zhangyuxin@cqu.edu.cn

## Abstract

In this work, a diatomite@graphene@ZnO (ZGD) photocatalyst was synthesized by chemical vapor deposition and hydrothermal methods and used for the photocatalytic degradation of methylene blue. The characterization of the prepared nanocomposite was performed by scanning electron microscopy (SEM), energy dispersive spectroscopy (EDS), X-ray photoelectron spectroscopy (XPS), Fourier transform infrared spectroscopy (FTIR), Raman spectroscopy, X-ray diffraction (XRD), and N_2_ adsorption–desorption techniques. Ultraviolet-visible diffuse reflectance spectroscopy (DRS) showed that the prepared ZGD photocatalyst enhanced the absorption of visible light and induced a red-shift. Photoluminescence spectroscopy (PL) revealed that the recombination of electron and hole pairs can be effectively suppressed. Besides, the synergistic effect of diatomite and graphene avoids the agglomeration of ZnO, increases the number of surface adsorption sites, and limits the electron transport, consequently improving the photocatalytic activity of ZnO. When ZGD-3 was UV-irradiated (*λ* = 663 nm) for 90 minutes, the degradation effectiveness of methylene blue (MB) was 100%. After the fifth repetition, the photocatalytic degradation efficiency was always greater than 95%. Simply put, the ZGD nanocatalyst can be used as an efficient photocatalyst for dye wastewater treatment.

## Abstract 2

In this work, a diatomite@graphene@ZnO (ZGD) photocatalyst was synthesized by chemical vapor deposition and hydrothermal methods and used for the photocatalytic degradation of methylene blue.

## Graphical abstract

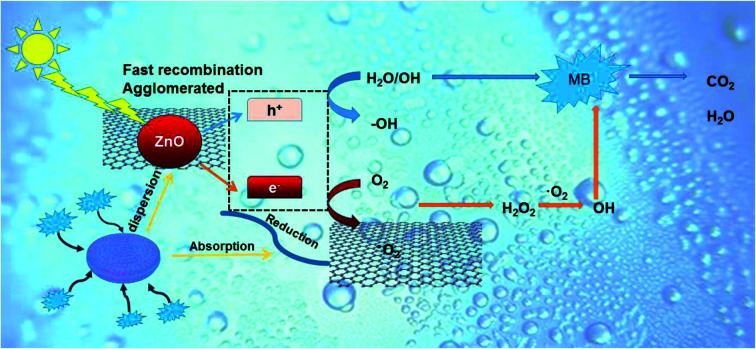

## 1. Introduction

Industrial production produces a substantial amount of pollution, seriously destroying the environment, and among them, dye wastewater has been a focus of attention in recent years.^1,2^ The dye wastewater produced by many industries, such as textile, plastic, clothing, leather, food processing, *etc.*, contains a large number of organic dyes.^3,4^ Dye wastewater has a high organic pollutant content and complex composition, exhibits resistance to photolysis and oxidation, and contains a variety of biotoxic or carcinogenic, teratogenic or mutagenic organic substances, which are extremely harmful to the environment.^5^ In particular, methylene blue (MB) is applied for a wide range of applications, such as chemical indicators, dyes, biological stains, and drugs, due to its exceptional staining effect and reducibility. Unfortunately, the high toxicity, stable structure, and deep color add to the difficult treatment of methylene blue wastewater.^6–8^ Some effective water purification strategies have been explored; for example, adsorption,^9^ filtration,^10^ and biodegradation^11^ are commonly used in methylene blue wastewater treatment. Among various environmental pollution treatment technologies, photocatalysis with semiconductor oxides as catalysts can deeply mineralize and decompose harmful substances and has become an ideal technology for dye wastewater pollution treatment.^12,13^

After years of investigation, semiconductor materials such as LaTiO_2_N,^14^ ZnO, Fe_2_O_3_,^15^ selenium-enriched amorphous NiSe1 + *x* nanoclusters,^16^ and CdS^17^ have been found to have photocatalytic functions and have received considerable attention. Among them, the ZnO photogenerated electron–hole pair binding energy can reach 60 meV,^18^ and it exhibits high photocatalytic activity, and has good photochemical stability, low cost and low toxicity, meaning that it is frequently used for the degradation of organic pollutants.^19–22^ However, ZnO has a wide bandgap (3.37 eV), so the photocatalytic electron–hole pairs can easily recombine, significantly reducing the quantum efficiency of photocatalysis, resulting in the low photocatalytic efficiency of ZnO.^23^ Additionally, the agglomeration of ZnO reduces the ideal specific surface area, resulting in lower reactivity. Moreover, direct use of nanoparticles in suspensions can lead to de-energization during catalysis, which causes severe degradation of the photocatalytic performance.^24^ Hence, ZnO in suspension must be immobilized on a suitable carrier before being used. Many materials, such as glass,^25^ montmorillonite,^26^ mesoporous silica,^27^ polyvinyl alcohol,^28^ electrodeposited tubes,^29^ and template esters,^30^ are commonly used as carriers or stabilizers, particularly by combining ZnO, with graphene being preferred. As a versatile carbon material, graphene has a two-dimensional conjugated chemical structure that enables it to possess appealing properties, like biocompatibility, high electrical conductivity, optical transparency, mechanical stability, and high thermal/chemical stability.^31–33^ The exceptionally large specific surface area allows graphene composites to absorb more pollutants. And the favorable electron mobility and carrier properties of graphene are favorable for the transport and separation of photoexcited charges, and are conducive to the inhibition of electron–hole pair recombination, making graphene/semiconductor composites have great promise in pollutant degradation. In previous research, the excellent properties of composites formed by the combination of ZnO and carbon-based materials can be seen.^34–42^

Diatomite is a natural three-dimensional porous biotemplate with non-toxicity, excellent mechanical properties, favorable corrosion resistance, and high adsorption properties.^43^ Similarly, its high specific surface area, uniformly distributed porous structure, and low cost make it an exceptional catalyst carrier material.^44–47^ The rich nano-pore structure of diatomite can provide a good composite anchor point for catalytic particles, which is conducive to the uniform dispersion of the catalytic particles and the reduction of inter-particle polymerization, increasing the number of active sites for the catalytic reaction, and improving the photocatalytic efficiency.^46^ Besides, diatomite-based photocatalytic materials^48–50^ frequently have excellent adsorption properties, which can effectively improve the contact probability between the catalyst and pollutants, while also helping to overcome the insufficient adsorption of pollutants due to the small specific surface area of the catalyst, thus improving the photocatalytic activity.

**Scheme 1:**
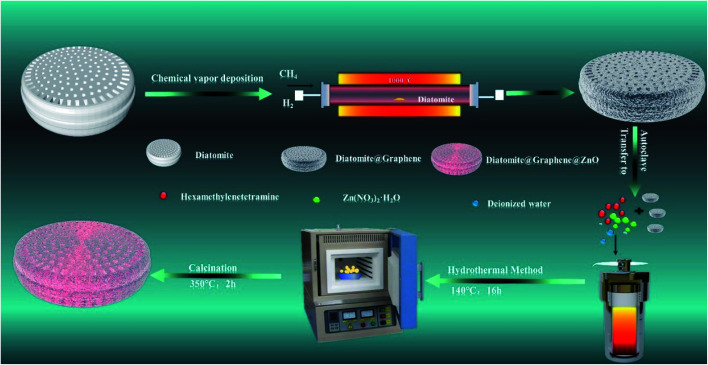
Schematic diagram showing the process of the synthesis of graphene@diatomite and diatomite@graphene@ZnO.

As depicted in the steps of Scheme 1, chemical vapor deposition was used to load graphene without any added functional groups directly onto diatomite. Then, ZnO was loaded by hydrothermal growth and calcination onto the diatomite@graphene. Under photocatalytic conditions, the diatomite@graphene@ZnO catalyzed the processing of water samples containing methylene blue. The present study reports a high-efficiency photocatalyst, explores the preparation of the composite material and the optimal reaction conditions, investigates the catalytic efficiency of different composition ratios in degrading methylene blue dye wastewater, and elucidates the catalytic mechanism.

## 2. Materials and experimental methods

### 2.1. Fabrication of diatomite@graphene

The chemicals were of analytical grade and used without further purification. Diatomite@graphene was synthesized by chemical vapor deposition. Initially, in a CVD tube furnace, the diatomite was heated from ambient temperature to 1000 °C under argon and hydrogen, and the heating time was 60 minutes. Thereafter, maintaining the argon and hydrogen supply, methane was introduced and reacted at 1000 °C for 120 minutes. Then the methane and hydrogen supplies were switched off, and the sample was cooled to 300 °C, and then cooled quickly. Diatomite@graphene powder labeled as GD was obtained.

### 2.2. Fabrication of diatomite@graphene@ZnO

0.28 g of hexamethylenetetramine and various weights (0.119 g, 0.283 g, 0.476 g, 0.982 g, and 1.904 g) of Zn(NO_3_)_2_·6H_2_O were dissolved in 35 mL of deionized water. The solution was then magnetically stirred for 20 minutes, poured into a 50 mL Teflon-lined stainless-steel autoclave containing 30 mg of diatomite@graphene and heated at a hydrothermal temperature of 140 °C for 16 hours. Then, the Teflon-lined stainless steel autoclave was allowed to cool naturally and the product was washed several times by centrifugation with deionized water and anhydrous ethanol. The obtained precursors were dried in an oven at 60 °C for 10 hours. Eventually, the dried samples were calcined in a 350 °C muffle furnace for 2 hours. Pure ZnO was also obtained under the same conditions as well. The diatomite@graphene@ZnO samples with different ZnO contents were labeled as ZGD-1, ZGD-2, ZGD-3, ZGD-4, and ZGD-5, respectively.

### 2.3. Photodegradation of MB

To evaluate the photocatalytic activity of the prepared samples in the photocatalytic degradation of MB, 20 mg of diatomite@graphene@ZnO at different ZnO contents was mixed with 100 mL of MB (*C*_0_ = 30 mg L^−1^) solution and magnetically stirred in the dark for 10 minutes to allow the MB solution to reach the adsorption–desorption equilibrium. A 10 watt UV lamp was used as a light source to degrade the MB solution. The UV lamp was placed approximately 20 cm from the surface of the solution. Aliquots of the solution were taken at regular intervals and the sample and liquid were separated by high-speed centrifugation. Under the same experimental conditions, no catalyst was added for the control experiment. The concentration of MB in the supernatant was then determined by assessing the absorbance intensity at 663 nm by UV-Vis spectrophotometer and the catalytic degradation rate of MB was estimated using the equation:1
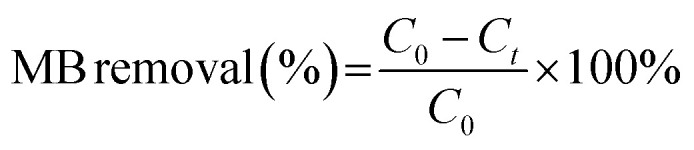
where *C*_0_ and *C*_*t*_ (mg L^−1^) are the liquid-phase concentrations of the dye at the initial point and time *t*, respectively. To examine the reusability of the catalyst, a repeatability test was conducted. Taking ZGD-3 as an example, after degradation, the mixture was centrifuged, filtered, and washed several times, and the residue was collected after drying. The cyclic test was repeated five times under the described photocatalytic condition parameters. The final concentration of MB dye solution after each catalytic test was estimated by the method described above.

### 2.4. Characterization

Analysis of the crystal information and chemical composition of the as-prepared samples was performed by X-ray diffraction (XRD) using a Rigaku D/max-2500 (Cu Ka radiation, *k* = 0.15406 nm) at a scanning speed (5 min^−1^) in the range of 5–80°. FIB/SEM (Zeiss Model Auriga) was used to evaluate the morphology and structure of the prepared samples. The Fourier transform infrared spectra (FT-IR) of the samples were recorded on a Nicolet iS5 spectrometer using the KBr disk method with a sample/KBr mass ratio of 1 : 100 within the range of 4000 to 400 cm^−1^. The surface element state analysis was recorded by X-ray photoelectron spectroscopy (XPS, Thermo ESCALAB 250Xi X-ray photoelectron spectrometer). The samples were detected by a LabRAM HR Evolution Raman spectrometer. N_2_ adsorption–desorption isotherms were determined by using a Micrometric instrument (ASAP 2020). The photocatalytic activity of the samples was measured by a UV-3600 UV visible near-infrared spectrophotometer.

## 3. Results and discussion

### 3.1. Characterization of diatomite@graphene@ZnO

**Fig. 1:**
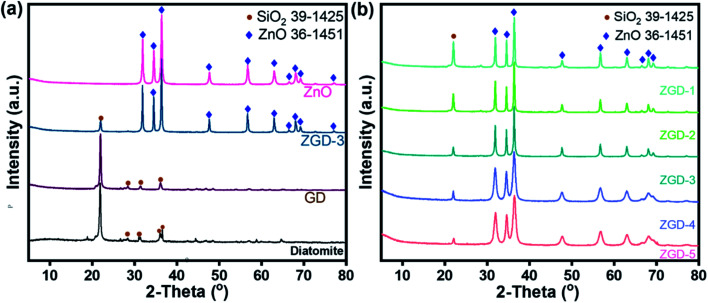
(a) XRD patterns of GD, ZGD-3, and ZnO; (b) XRD patterns of ZGD-1, ZGD-2, ZGD-3, ZGD-4, and ZGD-5.

Fig. 1a presents the XRD patterns of the pure ZnO, diatomite, GD, and ZGD-3 as prepared. The diffraction peaks at 22.0°, 28.4°, 31.5° and 36.1°, which are respectively assigned to the (101), (111), (102) and (200) facets, are consistent with SiO_2_ (JCPDS card no. 39-1425), indicating the presence of diatomite in the GD composites. In addition, the characteristic peaks of ZnO can be seen to occur at 2-theta = 31.8°, 34.4°, 36.3°, 47.5°, 56.6°, 62.9°, 66.4°, 68.0°, 69.1° and 77.0°, respectively assigned to the (100), (002), (101), (102), (110), (103), (200), (112), (201) and (202) facets, which are indexed to ZnO (JCPDS card no. 36-1451). Also, the XRD patterns of ZGD-3 showed the characteristic peaks of ZnO and diatomite. Furthermore, the crystallinity of the composites can be defined by the characteristics of the main diffraction peak. From Fig. 1b, an indication of the relative intensity of the main ZnO diffraction peaks can be used to determine the relative content of ZnO crystals in the samples. However, with the increase of the ZnO content, the efficiency of the photocatalytic degradation of methylene blue will become unsatisfactory, as a high crystallinity leads to the clogging of the diatomite pores by ZnO, which reduces its adsorption capacity and leads to the reduction of the electrical conductivity of graphene. Because diatomite's absorption and release of water in methylene blue solution can decompress the water molecules into positive and negative ions to encase the water molecules and form positive and negative ion groups, the catalytic effect is enhanced. Therefore, a suitable ratio should be found.

**Fig. 2:**
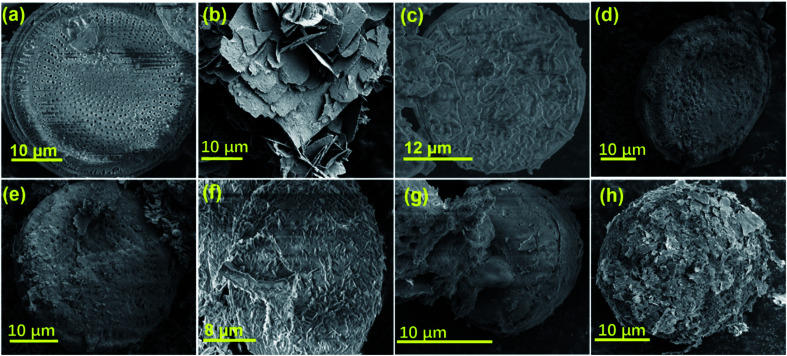
SEM images of (a) diatomite, (b) ZnO, (c) GD, (d) ZGD-1, (e) ZGD-2, (f) ZGD-3, (g) ZGD-4 and (h) ZGD-5.

The morphology and microstructures of diatomite, pure ZnO, and ZGD-1, ZGD-2, ZGD-3, ZGD-4, and ZGD-5 are shown in Fig. 2. Pure diatomite has a predominantly porous disc-like, highly mesoporous structure with a diameter of about 20 μm, as shown in Fig. 2a. The agglomeration of pure ZnO (Fig. 2b), not favorable for dispersion, seriously impacts its photocatalytic efficiency. Following the introduction of graphene, the SEM image of GD shows a well-defined film-like structure that avoids the agglomeration of diatomite and also increases the dispersal of graphene. The introduction of graphene also allows ZnO to be partially dispersed on the graphene, thus improving the dispersibility of ZnO. The SEM images depicted in Fig. 2d to h correspond to ZGD-1, ZGD-2, ZGD-3, ZGD-4, and ZGD-5, respectively. With the increase in ZnO content, the ZnO grown on the GD gradually increases, and ZGD-5 has been entirely wrapped by ZnO. On the contrary, the ZnO on the surface of the ZGD-3 sample is evenly distributed.

**Fig. 3:**
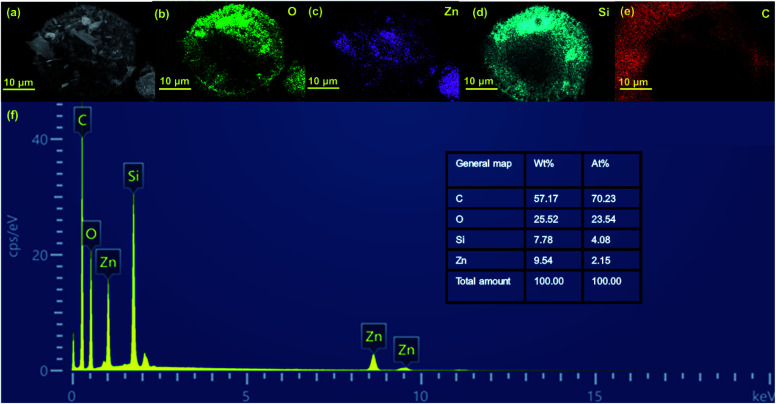
(a–e) The EDS mapping images of ZGD-3; (f) the EDS spectrum of ZGD-3.

Energy dispersive spectroscopy (EDS) mapping images indicate the coexistence of O, Zn, Si, and C elements in ZGD-3 (Fig. 3a–e), in which the elements of O and Si are attributed to diatomite, while Zn and O originate from the ZnO, and the C element is attributed to graphene. Moreover, the EDS mapping images demonstrate the spatial distribution of Zn on the GD surface without any noticeable segregation, indicating the successful growth of the ZnO on the GD surface. The purity of the product was confirmed by EDS analysis in Fig. 3f and the O elements in the sample.

**Fig. 4:**
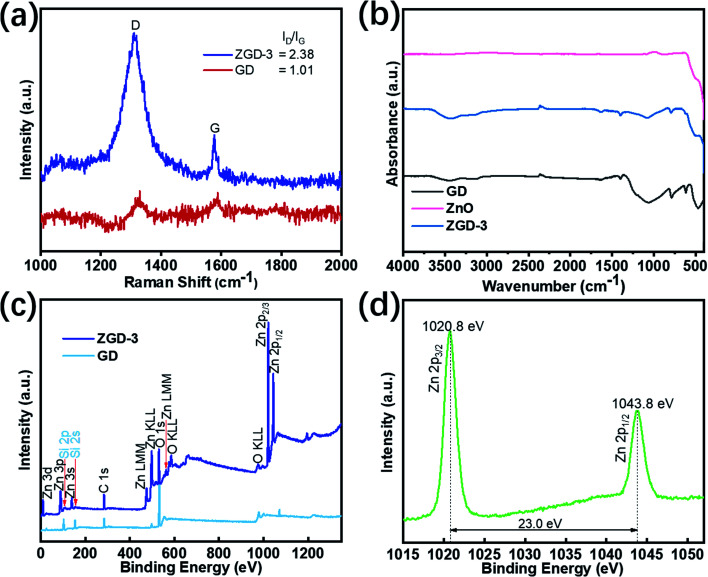
(a) The Raman spectra of GD and ZGD-3; (b) the FT-IR spectra of GD, ZnO, and ZGD-3; (c) the wide range XPS spectra of GD and ZGD-3; (d) the high-resolution spectrum of Zn 2p from ZGD-3.

The typical Raman spectra of ZGD-3 and GD are depicted in Fig. 4a. The Raman spectra of ZGD-3 and GD exhibit similar D and G bands of carbon, implying that the structure of GD is maintained in the ZGD-3. The D and G bands of graphene could cause the two remarkable peaks at 1350 cm^−1^ and 1590 cm^−1^, respectively. The G band corresponds to the in-plane vibration of sp^2^ to sp^3^ hybridized carbon, originating from the destruction of the sp^2^ structures of the graphite or due to the covalent attachment of functional groups. The D band is related to the disorder in the graphitic structure. Additionally, the intensity ratio of the D-band to G-band (*I*_d_/*I*_g_) indicates the degree of structural defects in graphene materials. Compared with GD (1.01), the intensity ratio of the D-band to G-band (2.38) of ZGD-3 is also enhanced, indicating that the disorder of the graphene sheets is improved. Notably, the Raman peaks of ZD and ZGD-3 shifted somewhat. These shifts in the Raman peaks may be due to chemical interactions between ZnO and graphene.^22^

Fig. 4b presents the FT-IR spectra of ZGD-3, pure ZnO, and GD. The peak of GD at 3445.53 cm^−1^ is formed by the free silanol group (SiO–H) on the surface. This peak shifted from 3445.53 cm^−1^ to 3420.82 cm^−1^ with the growth of ZnO. Furthermore, the peaks located at 1645.43, 1399.52, 1062.02, 788.45, 616.87 and 467.66 cm^−1^ were assigned to the presence of the (H–O–H) bending vibration of water retained in the diatomite, asymmetric stretching modes of the Si–O–Si bond, Si–O stretching of the silanol group, and Si–O–Si bending vibration of the diatomite structure, respectively. In the GD and ZGD-3 samples, the stretching vibration peak of C=C of the graphite skeleton is at 1635 cm^−1^, and there is no absorbance peak of a carbonyl group or epoxy group, which indicates that the graphene does not contain functional groups. When ZnO was successfully compounded, the intensity of these peaks decreased, indicating that ZnO was successfully *in situ* grown on the diatomite@graphene.

X-ray photoelectron spectroscopy (XPS) measurement was carried out to evaluate the elemental composition of GD and ZGD-3 as shown in Fig. 4c. The resulting data were corrected with the binding energy of 284.6 eV of exogenous carbon. The wide range of the XPS spectra indicates the presence of Zn, C, O, and Si, demonstrating the successful loading of ZnO onto the GD. The subsequent high-resolution XPS spectrum obtained for Zn2p is shown in Fig. 4d. At the energy position of 1015–1055 eV, two peaks are detected corresponding to the Zn2p3/2 and Zn2p1/2 orbitals with energies of 1020.8 eV and 1043.8 eV, respectively.

**Fig. 5:**
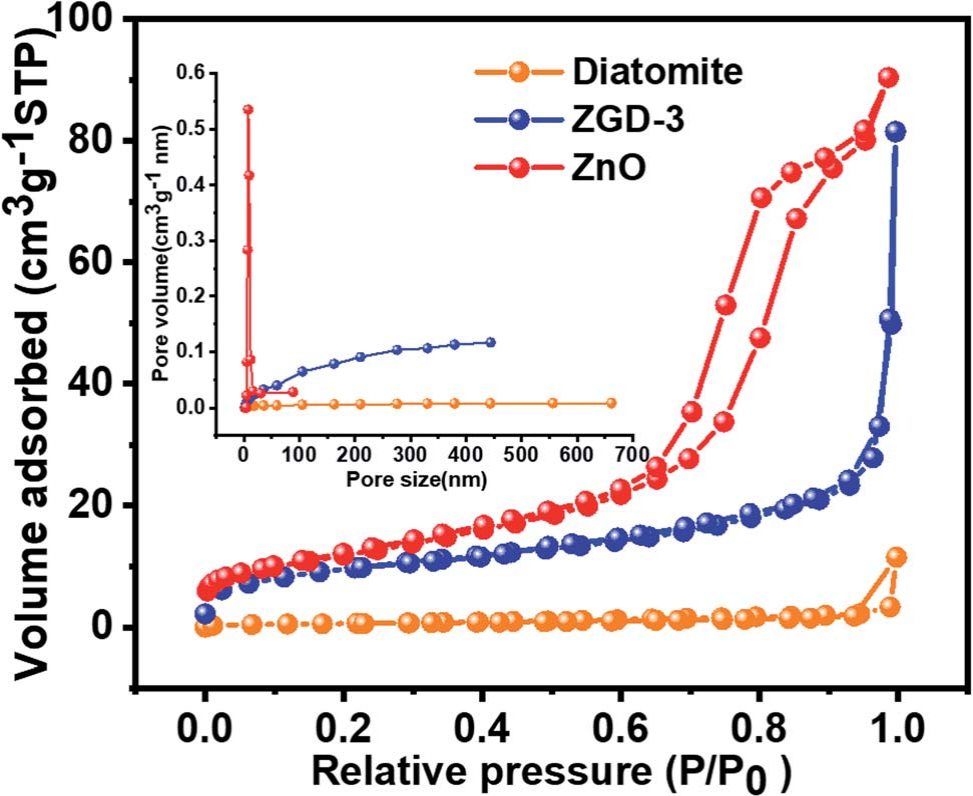
The N_2_ adsorption–desorption isotherms and pore-size distribution (inset) of diatomite, ZGD-3, and ZnO.

Nitrogen adsorption/desorption isotherms were used to analyze the specific surface area of the samples and the pore size distribution, as shown in Fig. 5. According to the IUPAC classification, diatomite, ZGD-3, and ZnO all exhibited reversible type IV isotherms, indicating that the materials are dominated by mesoporous structures internally. The specific surface areas of diatomite, ZGD-3, and ZnO were 2.4078, 34.644, and 43.235 m^2^ g^−1^ respectively. The pore sizes of diatomite, ZGD-3, and ZnO were 10.131, 12.293, and 7.840 nm, respectively. The pore sizes of diatomite and ZGD-3 were mainly distributed at 200 to 300 nm, and the pore size of pure ZnO was mainly divided negatively at 5 to 20 nm.

As a consequence of these results, it could be confirmed that the diatomite@graphene@ZnO composites were successfully synthesized.

### 3.2. The effect of synthetic parameters

#### 3.2.1. The effect of hydrothermal synthesis temperature on the crystal structure of diatomite@graphene@ZnO

To further investigate the effect of experimental parameters on the controllable preparation of ZGD, the following experiments were conducted. The hydrothermal reaction temperature is an influential factor in the crystal structure of composites. Experiments were carried out at different reaction temperatures (120 °C, 140 °C, 160 °C) for the same reaction time to determine the influence of reaction temperature. As seen in Fig. S1(a),† the peak of diatomite decreases and then increases with increasing reaction temperature and is smallest at 140 °C. The peak of ZnO exhibits no significant change, which demonstrates that temperature has less influence on its crystallinity. Combined with the electron micrographs in Fig. S2(a)–(c),† all of which show ZnO wrapped around the GD, it can be seen that at 120 °C, some of the ZnO is not completely grown, so there is a vacant hole. At 160 °C, therefore, the ZnO lamellae are overgrown, resulting in their inability to be coated with diatomite@graphene for loading. In comparison, at 140 °C, the ZnO loading was uniform and well-shaped, so the temperature of 140 °C was more favorable for the preparation of ZGD.

#### 3.2.2. The effect of hydrothermal synthesis time on the crystal structure of diatomite@graphene@ZnO

Hydrothermal synthesis time is also an essential factor affecting the crystallographic change of ZGD. Samples were collected at different times (12 h, 16 h and 24 h) at a reaction temperature of 140 °C, and then washed, dried and calcined to obtain ZGD. From the electron microscopy image in Fig. S2(a),† it can be seen that the ZnO failed to load entirely on the GD at the reaction time of 12 h. Furthermore, from Fig. S1(b),† the peak of diatomite reached its lowest at 16 h. As the reaction time expanded to 24 h, the peak of diatomite increased and the peak of ZnO weakened. Combined with Fig. S3(c),† the hydrothermal synthesis time of 24 h resulted in the shedding of ZnO from the surface of GD. This demonstrates that too long a time is not conducive to the synthesis. In contrast, at 16 h, the sample had good morphology and no ZnO peeling was observed, so this time was most preferable for the synthesis of the sample.

### 3.3. Evaluation of photocatalytic activity

**Fig. 6:**
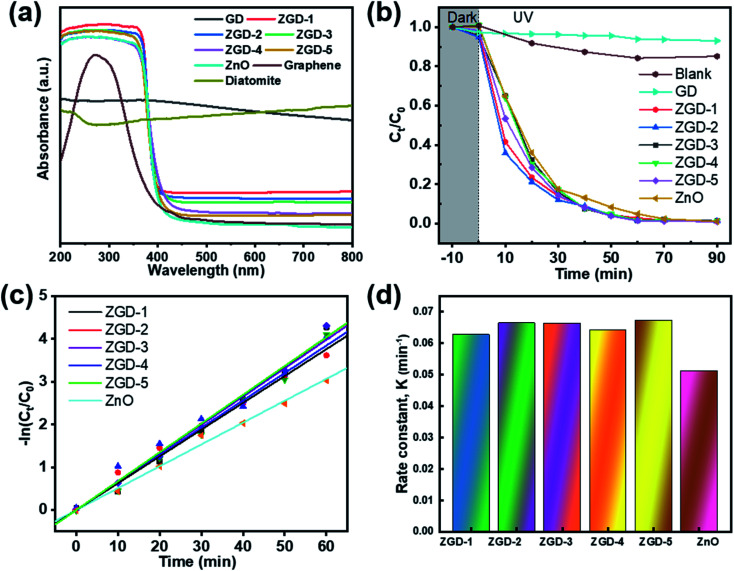
(a) UV-vis diffuse adsorption spectra of diatomite, graphene, GD, ZGD-1, ZGD-2, ZGD-3, ZGD-4 and ZnO. (b) The photocatalytic degradation of MB in the presence of GD, ZGD-1, ZGD-2, ZGD-3, ZGD-4, and ZnO. (c) The kinetic plots of the degradation of MB under different reaction conditions. (d) Photodegradation rate constants of the photocatalysts.

As demonstrated in Fig. 6a, the optical properties of the GD and the ZGD with different ZnO contents were tested using UV-Vis diffuse reflectance spectroscopy (UV-Vis). The ZnO emitted a characteristic spectrum with a basic absorption sharp edge rising at 400 nm. The UV characteristic absorption peak of graphene is at about 270 nm. The adsorption edge of the ZGD sample was similar to that of ZnO when the GD was loaded with ZnO. Furthermore, there was a clear correlation between the ZnO content and the change in the UV-Vis spectrum. The adsorption performance increased with increasing ZnO content, and none of the bandgap energies of the prepared samples changed, with strong, broad background absorption in the visible region of the UV-Vis spectrum. These observations may indicate an increase in the surface charge of the oxide in the ZGD.^37^

In the photocatalytic degradation process, the dye is first adsorbed on the catalyst surface, and the catalyst generates electron–hole pairs under light conditions so that the dye molecules escape as a gas. The degree of photodegradation is determined by the amount of adsorption and the separation or recombination of electrons and holes.^24,51^ According to Fig. 6b, the degradation rate of MB over ZGD with different contents of ZnO is higher than that over pure ZnO and GD under ultraviolet light. The degradation rate of MB over GD firstly increases and then decreases, which is due to the porous structure of graphene@diatomite. The MB solution could not be degraded by adsorption on GD and was released after saturation, reducing the self-degradation rate of the MB solution under UV light. From Fig. 6c, the photocatalytic performance of diatomite@graphene@ZnO greatly exceeded that of pure ZnO, indicating that the synergistic effect of diatomite and graphene promoted the photocatalytic efficiency. On the one hand, the effective separation of graphene by diatomite as a natural 3D biotemplate significantly improves the dispersion of graphene. On the other hand, the *in situ* growth of ZnO on graphene leads to enhanced photocatalytic activity, which can be attributed to the inhibition of electron–hole pair binding and the increased efficiency of electron–hole pair separation due to fast photoinduced charge separation. The pseudo-first order kinetics with respect to the concentration of MB of ZnO at 60 minutes is 3.2 , significantly lower than the value for the introduction of diatomite@graphene. It was revealed that the introduction of diatomite and graphene played an essential function in facilitating the photocatalytic ability of ZnO.

The Langmuir–Hinshelwood model can be used to express the photocatalytic reactions on the ZGD surface.^51^ Pseudo-first-order kinetics was applied to the photocatalytic degradation of MB by the ZGD.2
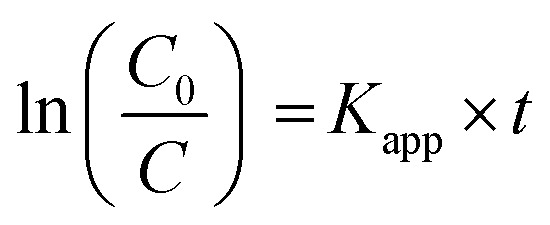
where *K*_app_ is the apparent reaction rate constant, used as the basic kinetic parameter for different photocatalysts; *C*_0_ is the initial concentration of MB in aqueous solution at time *t* = 0, and *C*_*t*_ is the residual concentration of MB at time *t*. The results are presented in Fig. 6d, which shows that the apparent rate constant for ZnO is 0.05109 min^−1^. The apparent rate constant for ZGD-3 is 0.06638 min^−1^.

**Fig. 7:**
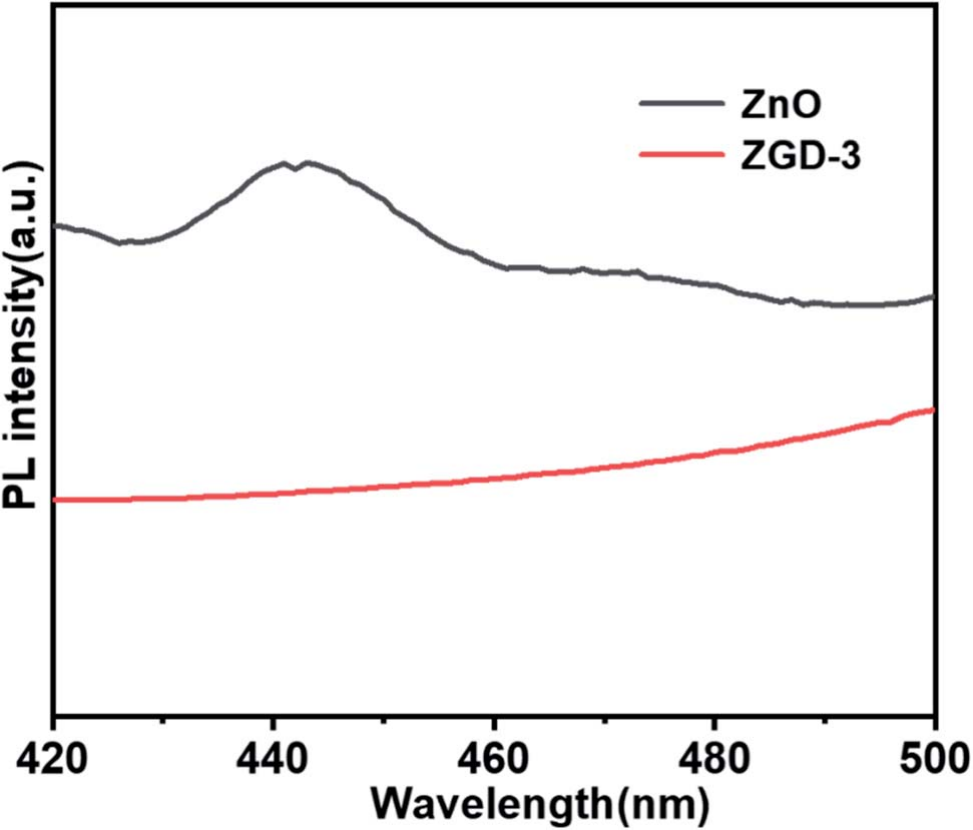
Photoluminescence spectra of ZnO and ZGD-3.

Fig. 7 reveals the photoluminescence spectroscopy (PL) spectra of the pure ZnO and ZGD-3. For the pure ZnO, the PL spectrum exhibits a strong emission peak at 442 nm, which originates from the band-to-band recombination of electrons and holes. The PL spectrum demonstrates that the intensity of ZGD-3 was significantly reduced, confirming that the recombination of photo-induced charge carriers can be inhibited and the photocatalytic degradation performance of ZnO was enhanced. To further elucidate the degradation of methyl blue by ZGD-3 compared with other reported catalysts, Table 1 lists several composite nanomaterials and a comparison shows that the materials in this paper have exceptional performance in the photocatalytic degradation of MB.

**Table 1:** Photocatalytic degradation of MB by the novel composite

Nanocomposite material	Photo-catalytic efficiency/%	Degradation time/min	Reference
ZnO/graphene	100	180	52
ZnO/C	100	100	53
ZnO/CMOF-5	99	360	54
TiO_2_/GO	98.67	45	55
Sr/TiO_2_	100	80	56
Pt/ZnO	100	60	57
CQDs/meso-Ti-450	98	60	58
CuCr_2_O_4_/CuO	95	120	59
ZGD-3	100	90	This work

### 3.4. The stable reusability of diatomite@graphene@ZnO

**Fig. 8:**
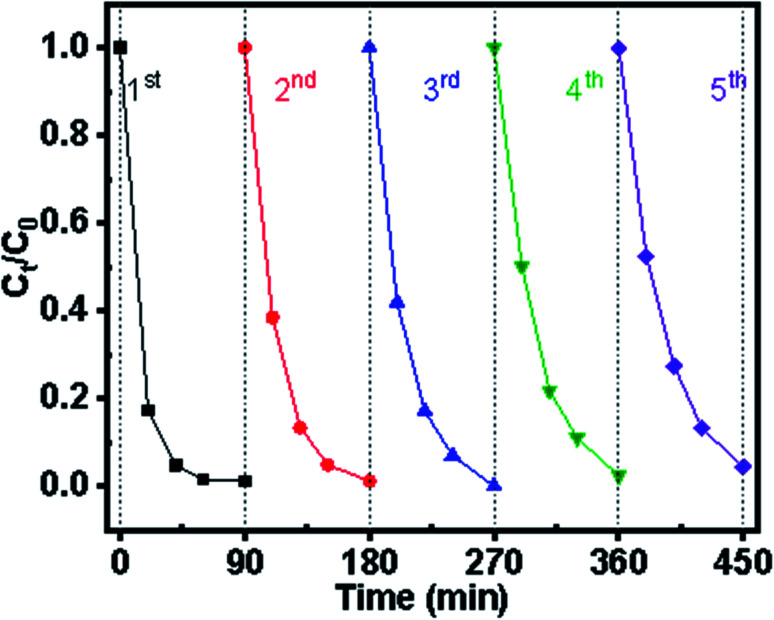
The reusability experiment results of diatomite@graphene@ZnO.

Catalytic stability has been the limiting factor for long-term application in practical wastewater treatment. Using ZGD-3 as the investigated sample, the experiment was cycled five times, each time with 90 min of UV light. In the first photocatalytic cycle, the degradation rate of MB was 100%, displaying excellent photocatalytic performance. From Fig. 8, it can be seen that ZGD-3 showed a slow decrease in photocatalytic rate as the number of cycles increased. The reason for the decrease in decolorization rate was that some by-products of the targeted pollutants were adsorbed within the catalyst pores, reducing the number of active sites of OH^−^. Nevertheless, after the 5th repetition, the decolorization rate was still above 95% and the effect on the amount of degradation was not significant.

### 3.5. Mechanism of photocatalytic degradation

**Fig. 9:**
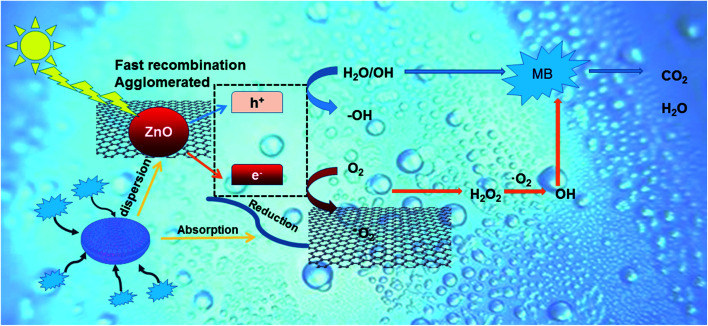
Proposed mechanism for the photodegradation of MB by diatomite@graphene@ZnO.

The higher photocatalytic performance may be achieved by the joint action of the adsorption and separation of diatomite and the recombination inhibitor effect of graphene. A proposed mechanism for the photodegradation of MB by ZGD is described in Fig. 9. The photoinduced electron–hole pairs could be generated under UV light irradiation. In previous experiments, carbon-based materials, such as GO, RGO, and graphene, played the role of both electron-transporters and electron-acceptors, and the carbon-based materials that we mentioned will promote the migration of photoinduced electrons and block charge recombination to enhance the photocatalytic performance of ZnO.^34–38^ The photogenerated electrons will be transferred to the graphene since the energy level of graphene is lower than the conduction band of ZnO, and this process effectively inhibits the photoelectron–hole pair recombination. Then, the electrons will react with O_2_ to generate ˙O^2−^, and ˙O^2−^ can degrade MB into CO_2_, H_2_O, and other inorganic substances. In addition, more ˙O^2−^ can be generated on the surface of graphene due to its larger surface area.

The advantages of using graphene are as follows: restricted charge carrier recombination, the increase of surface adsorption sites, the advantage of zero bandgap and π-conjugated structures,^60^ which achieves faster charge migration and so can efficiently boost the photocatalytic performance of ZnO. At the same time, photogenerated holes can react with H_2_O to generate strong oxidizing ˙OH, and ˙OH can effectively degrade MB. Moreover, when MB is captured on the surface of ZGD, the holes can also degrade it directly. Therefore, diatomite as a 3D template significantly increased the adsorption capacity of MB and effectively promoted the contact probability between the catalysts and pollutants. The rich nanopore structure of diatomite facilitates the homogeneous dispersion of graphene and ZnO, which as a carrier is actually equivalent to a solid dispersant, ensuring more exposure of catalyst active sites and boosting the transfer and separation of photoinduced carriers.^61^ Thus, the template effect of diatomite enhances the stability of the catalyst and facilitates secondary recycling. The complete mechanism can be described as follows.^62^ZnO + *hv* → e_CB_^−^ + h_VB_^+^H_2_O + h_VB_^+^ → ˙OH + H^+^˙OH + dyes → H_2_O + CO_2_O^2^ + e_CB_^−^ → O^2−^O^2−^ + dyes → H_2_O + CO_2_

## 4. Conclusion

In summary, diatomite@graphene@ZnO was successfully synthesized by using diatomite and graphene as a support, and the results were characterized by XRD, BET, XPS, Raman, SEM, DRS, and PL. The results showed that the reaction time and the hydrothermal temperature had significant effects on the structure of the synthesized samples. Furthermore, the prepared diatomite@graphene@ZnO is a highly efficient catalyst for the photocatalytic degradation of MB. The results of MB degradation showed that the degradation of diatomite@graphene@ZnO under UV irradiation was favorable, and the degradation rate was about 100% within 90 min. After 5 repetitions, diatomite@graphene@ZnO showed good reusability and is expected to become a simple and cost-effective alternative to wastewater treatment.

## Conflicts of interest

There are no conflicts to declare.

## Supplementary Material

RA-011-D1RA07708B-s001
