# Supplementary material for: 2D–3D graphene-coated diatomite as a support toward growing ZnO for advanced photocatalytic degradation of methylene blue
Source: RSC Adv. 2021 Nov 30;11(61):38505–14. doi: 10.1039/d1ra07708b (PMC9044178; doi:10.1039/d1ra07708b)
Supplement: RA-011-D1RA07708B-s001 [file RA-011-D1RA07708B-s001.pdf]

Electronic Supplementary Material (ESI) for RSC Advances.
This journal is © The Royal Society of Chemistry 2021

2D-3D magnetic NiFe layered double hydroxide decorated biotemplate as bifunctional material for simultaneously anionic and cationic dyes adsorption

Xingjian Dai[1], Hao Zeng[2], Chuan Jin[3], Jinsong Rao[4], Xiaoying Liu[5], Kailin Li[6], Yifan Zhang[7], Yaolun Yu[8], Yuxin Zhang[9] *

1 College of Materials Science and Engineering, Chongqing University, Chongqing, PR China 400044; Daixingjian@cqu.edu.cn

2 College of Materials Science and Engineering, Chongqing University, Chongqing, PR China 400044; 1196942050@qq.com

3 College of Materials Science and Engineering, Chongqing University, Chongqing, PR China 400044; 2972513971@qq.com

4 College of Materials Science and Engineering, Chongqing University, Chongqing, PR China 400044; likailin920809@163.com

5 Qian Xuesen Laboratory of Space Technology, China Academy of Space Technology, Beijing, 100094, China; yuyaolun@qxslab.cn

6 College of Materials Science and Engineering, Chongqing University, Chongqing, PR China 400044; zhangyuxin@cqu.edu.cn

**Correspondence: E-mail: zhangyuxin@cqu.edu.cn; Tel: 862365104131*

Supporting information

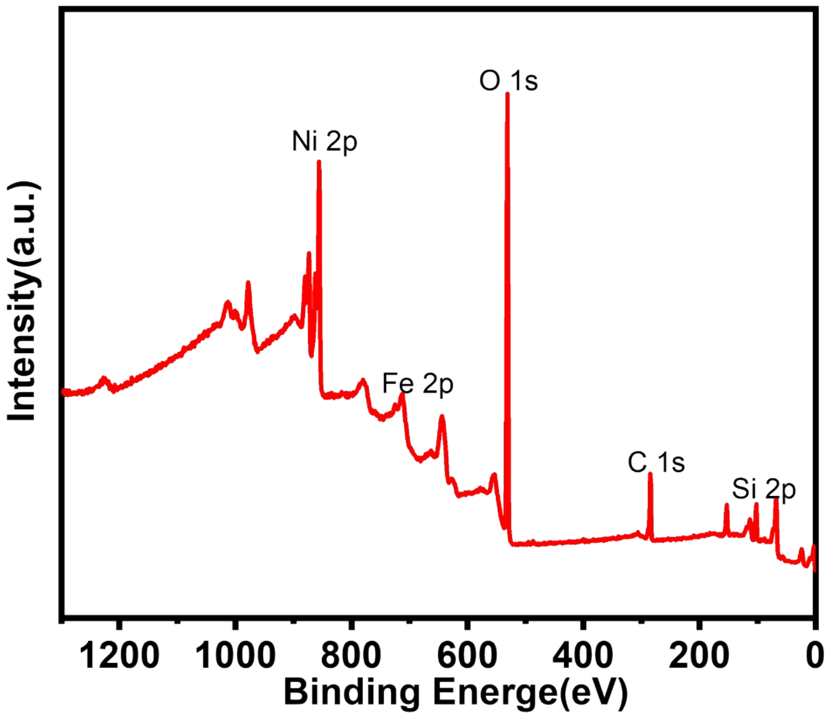

Fig.S1. XPS survey spectrum of DE@Ni_4Fe_1 LDH

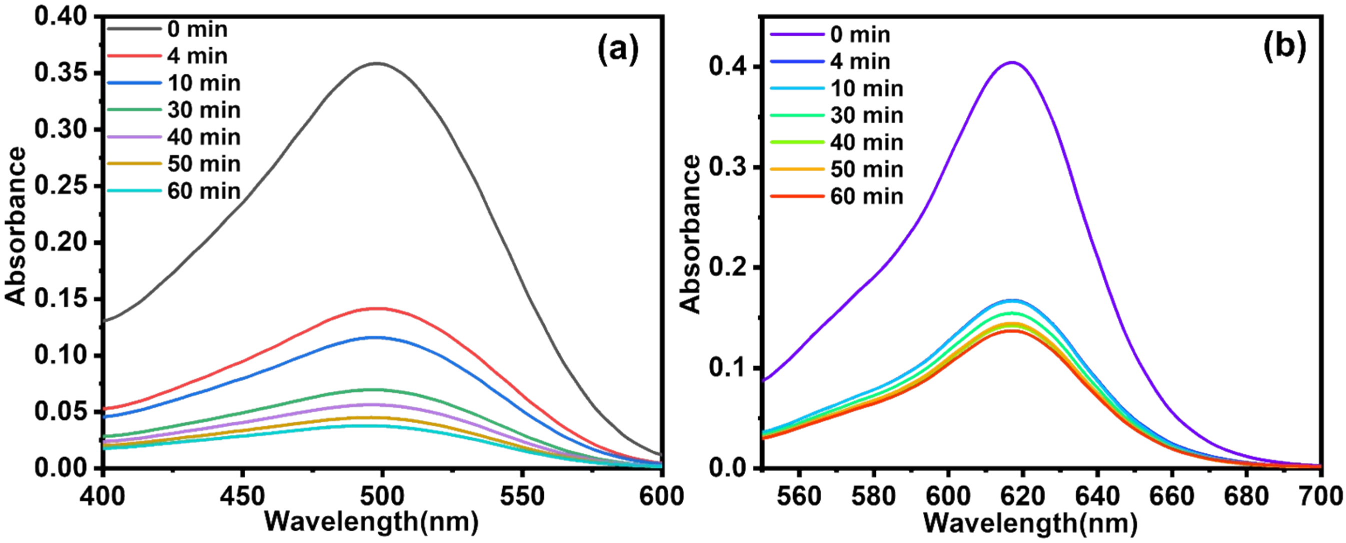

Fig.S2. UV-Vis absorption spectra of 20 mg L^{-1} (a) congo red, (b) malachite green after adsorption by DE@Ni_4Fe_1 LDH with different contact times.

Table S1. Characterization data of the as-synthesized DE@NiFe LDH.

Sample	d $_{(0\ 0\ 3)}$ (nm)	d $_{(1\ 1\ 0)}$ (nm)	c (nm)	a (nm)	σ(nm^2)
**DE@Ni_2Fe_1**	**0.755**	**0.154**	**2.266**	**0.307**	**0.041**
**DE@Ni_3Fe_1**	**0.764**	**0.154**	**2.291**	**0.307**	**0.027**
**DE@Ni_4Fe_1**	**0.771**	**0.1354**	**2.312**	**0.307**	**0.020**
